# Carboplatin in combination with weekly Paclitaxel as first-line therapy in patients with recurrent/metastatic head and neck squamous cell carcinoma unfit to EXTREME schedule

**DOI:** 10.18632/oncotarget.25157

**Published:** 2018-04-24

**Authors:** Adeline Pêtre, Cécile Dalban, Andy Karabajakian, Eve-Marie Neidhardt, Pierre Eric Roux, Marc Poupart, Sophie Deneuve, Philippe Zrounba, Jérome Fayette

**Affiliations:** ^1^ Centre de Lutte Contre le Cancer Léon Bérard, Lyon-I University, Lyon, France

**Keywords:** head and neck cancer, recurrence, metastasis, first-line, unfit

## Abstract

The standard first-line treatment in recurrent/metastatic head and neck squamous cell carcinoma combines Cisplatin, 5 Fluorouracil and Cetuximab, but many patients aren’t eligible. We retrospectively evaluated the efficacy and the tolerability of Carboplatin and Paclitaxel in this indication, mostly in patients unfit to Cisplatin.

Paclitaxel (80mg/m2) was administered at day 1, 8 and 15 and Carboplatin area under the curve 5 at day 1, repeated every 28 days, for 6 cycles. Carboplatin could be administered at area under the curve 2 at day 1, 8 and 15.

117 patients received this association at our institution, 94 of those were ineligible to cisplatin due to severe comorbidities, age >70years or Performance status >1.

The overall response rate was 40%. The median progression free survival for patients ineligible to Cisplatin was 4.4 months [95% CI; 3.4; 5.0] and the median overall survival was 8 months [95% CI; 5.4–10.7].

The most frequent toxicities were hematologic, with 94 grade ≥ 3, mostly in patients who received monthly Carboplatin.

Our study shows Carboplatin and Paclitaxel in first-line in recurrent/metastatic head and neck squamous cell carcinoma appear efficient for patients ineligible to Cisplatin and safe when both drugs are weekly administered.

## INTRODUCTION

Head and neck cancer accounts for 500,000 new cases and nearly 300,000 deaths annually worldwide [1] .

Squamous cell carcinoma is the most frequent histological subtype of head and neck tumors [2].

About two thirds of head and neck squamous cell carcinoma patients are diagnosed with locally advanced disease [3] and are treated with a combination of surgery, radiation therapy and chemotherapy. Despite this primary treatment, more than a third of these patients have locoregional recurrences or distant metastases [4–7] and in this situation treatment is mostly palliative and disappointing, with a median overall survival (OS) of less than 1 year [8].

About 10% of the patients present with distant metastases at diagnosis [9]. The most frequent site of distant metastasis is the lung [3].

Platinum based chemotherapy is the usual first-line palliative treatment. Currently, the standard of care is the combination of Cetuximab, Cisplatin and 5-Fluorouracil, followed by maintenance with Cetuximab (the “EXTREME” regimen) [10] .

A large proportion of patients are ineligible to the EXTREME regimen because of their age>70yo, Performance Status (PS)>1 or their severe comorbidities (cardiac or renal insufficiency etc…). In this population, despite replacement of Cisplatin with Carboplatin in the EXTREME regimen, tolerability remains poor.

In advanced non-small cell lung cancer, the standard of care is platinum based chemotherapy too. In elderly population, Carboplatin with weekly Paclitaxel is a safe and recommended option [11, 12].

Similarly to this indication in lung cancer, we propose in our institution Carboplatin with weekly Paclitaxel for patients unfit to Cisplatin and in some patients eligible to Cisplatin who refuse the EXTREME regimen for various reasons (mostly due to the 4 days of continuous perfusion of Fluorouracil).

This association isn’t validated in head and neck cancer but several studies have shown the efficacy of Paclitaxel as monotherapy or in combination with Cisplatin [13] with promising results.

In this retrospective study we evaluated the efficacy and the tolerability of Carboplatin and Paclitaxel as first-line treatment in patients with recurrent or metastatic head and neck squamous cell carcinoma (RMHNSCC), mostly for patients who are unfit to the EXTREME regimen.

## RESULTS

### Patients’ characteristics

Between August 2009 and December 2016, 117 patients with RMHNSCC were treated at the “Centre Léon Bérard “(Lyon, France) with Carboplatin and Paclitaxel combination as first-line therapy.

Patients’ characteristics are summarized in Table 1.

**Table 1:** Baseline demographics of the patient population

	*N* (%)
Median age, years [range]	66,2 [27.1–94.3]
Sex	
Female	25 (21)
Male	92 (79)
Localization at initial diagnosis	
Oral cavity	31 (26)
Oropharynx	33 (28)
Hypopharynx	25 (21)
Larynx	21 (18)
Other localization	6 (5)
Unknown	1 (1)
Tumor stage at initial diagnosis	
I	7 (6)
II	22 (19)
III	36 (31)
IV^a^	23 (20)
IV^b^	7 (6)
IV^c^	14 (12)
Unknown	8 (7)
Initial treatment	
Neoadjuvant chemotherapy	31 (26)
Platinium based	28 (24)
Unknown	3 (3)
Surgery	83 (71)
Radiotherapy	85 (73)
Alone	44 (38)
With Cisplatin	26 (23)
With Cetuximab	11 (9)
Other or unknown	4 (3)
Relapse before the introduction of Carboplatin+Paclitaxel	
Loco Regional only	57 (49)
Loco Regional and Metastatic	35 (30)
Metastatic only	13 (11)
Performance status at onset Carboplatin and Paclitaxel	
0	9 (8)
1	57 (49)
2	26 (22)
3	12 (10)
Unknown	13 (11)

Since 89% of patients experienced a recurrence, 41% received a platinum-agent previously for a localized disease (18% of those less than 6 months before relapse). Before palliative chemotherapy onset, 34 (29%) patients were treated locally for previous relapses (mainly surgery, radiotherapy potentiated or not by chemotherapy).The most common site of distant metastases was pulmonary .

All patients received Carboplatin and Paclitaxel strictly in first intent except for 8 patients. 2 of these patients should be retrospectively considered as initially metastatic: they had non-specific lung micronodules when they started induction chemotherapy with Cisplatin Docetaxel and Fluorouracil. After that they were treated with radiotherapy and one of them potentialized with Cisplatin. After 7.8 and 10 months of the last platin administration, the lung nodules progressed and they started carboplatin and paclitaxel. Despite a certain frailty, 5 patients received a first cycle of cisplatin-based chemotherapy and switched to Carboplatin and Paclitaxel due to severe toxicity. Finally 1 patient received a cycle of Cisplatin/Vinorelbine since the solitary metastasis was considered as a primary lung cancer and switched at the second cycle for Carboplatin with Paclitaxel.

Of the 117 patients treated with Carboplatin and Paclitaxel, 23 (20%) were eligible to a chemotherapy by Cisplatin Fluorouracil and Cetuximab (EXTREME regimen). 3 of those received firstly a Cisplatin-based chemotherapy, then, due to toxicity, Carboplatin and Paclitaxel. 94 (80%) were ineligible to EXTREME due at least to one adverse criteria among age >70 years, renal failure (creatinine clearance <60 ml/min), PS≥2 or severe comorbiditie(s) (cardiac insufficiency, cirrhosis, cisplatin allergy etc…). Details of ineligibility are summarized in Table 2.

**Table 2:** Frailty criteria of the 94 patients ineligible for the EXTREME schedule

	*N* (%)
All criteria listed :	
Age>70 yo	47 (40%)
Creatinine clearance <60ml/min	24 (21%)
Severe Comorbidities	29 (25%)
PS ≥2	38 (32%)
Patients unfit for cisplatin	94
Patients with only one criteria :	70 (74%)
Age	30 (26%)
Renal failure	2 (1%)
Severe comorbidities	14 (12%)
PS ≥2	24 (21%)
Patients with ≥2 criteria	21 (18%)
Patients with 1 criteria previous listed and another criteria :	3 (3%)
Lung cancer associated	2 (2%)
High dose of corticosteroids contraindicating the inclusion in a protocol	1 (1%)

### Carboplatin and paclitaxel delivery

**Figure 1:**
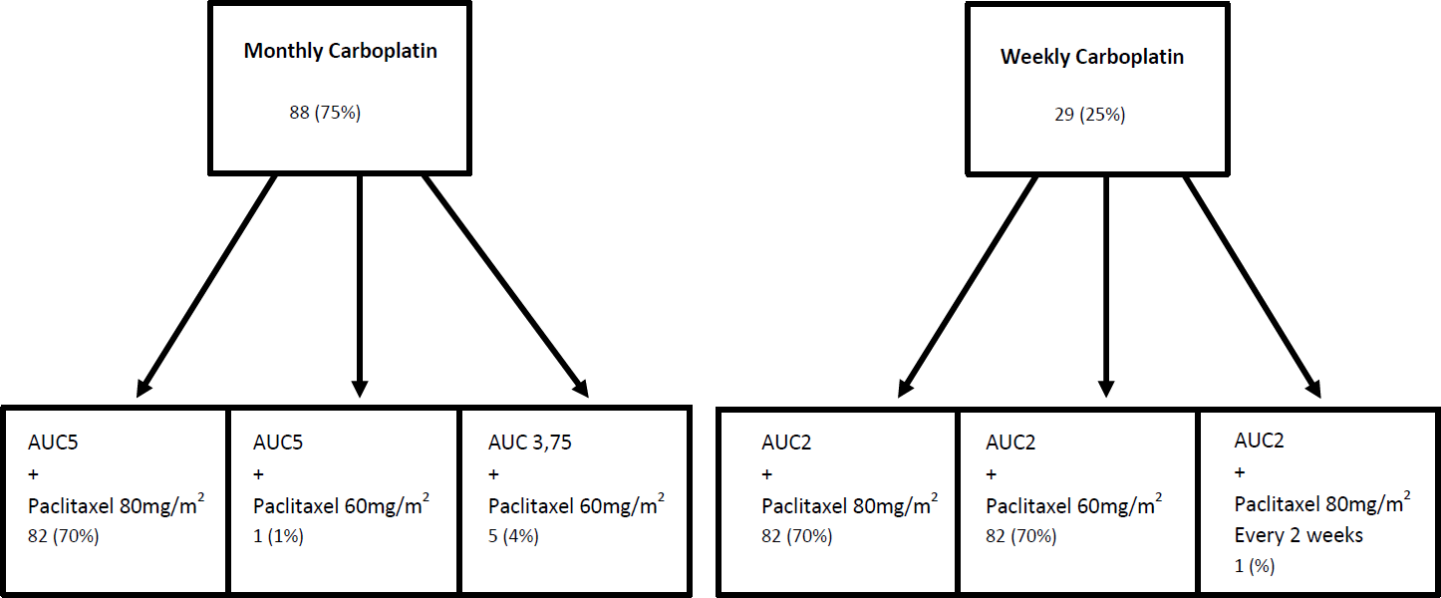
Delivery of carboplatin and paclitaxel

Data on chemotherapy delivery are summarized in Figure 1. Initially, Carboplatin was administered on a monthly basis for 88 (75%) patients and weekly for 29 (25%) patients. Due to severe frailty, 35 patients received a 25% reduction of the dose of a least one agent. Of note, 3 patients received Cetuximab in combination with carboplatin and paclitaxel. Treatment toxicities are presented in Table 3.

**Table 3:** Treatment toxicities

	Monthly Carboplatin *N* (%)	Weekly Carboplatin *N* (%)
Total of toxicities	88	40
Any grade toxicity leading to modification of treatment		
Dose reduction	53 (60%)	7 (18%)
With modification of monthly Carboplatin in weekly Carboplatin	11 (13%)	
Cessation of treatment	16 (18%)	9 (23%)
Anemia	45 (51%)	10 (25%)
≥grade 3)	23 (26%)	1 (3%
Thrombopenia	26 (30%)	8 (20%)
≥grade 3	7 (8%)	0 (0%)
Neutropenia	46 (52%)	20 (50%)
≥grade 3	35 (40%)	11 (28%)
Febrile neutropenia	12 (14%)	5 (13%)
Leading death	1 (1%)	1 (3%)
Infection without aplasia	39 (44%)	14 (35%)
Digestive toxicity	17 (19%)	5 (13%)
≥grade 3	1 (1%)	0 (0%)
Neuropathy	10 (11%)	3 (8%)
≥grade 3	2 (2%)	1 (3%)

Dose reduction was needed for 60 (51%) patients, 11 of those resulted with a modification of monthly Carboplatin to weekly Carboplatin and 25 (21%) stopped treatment due to toxicity.

The most common side effects were hematologic, they are detailed in Table 4. There were 94 grade ≥ 3 toxicities with 17 (15%) febrile neutropenia, 2 of those were fatal (2%). 18 patients needed granulocyte colony stimulating factors.

**Table 4:** Hemato toxicities

	Monthly Carboplatin					Weekly Carboplatin				
	1	2	3	4	5	1	2	3	4	5
Anemia	1	21	23	0	0	0	9	1	0	0
Thrombopenia	11	8	4	3	0	3	5	0	0	0
Neutropenia	1	10	26	9	0	1	8	9	2	0
Febrile neutropenia			5	6	1			4	0	1

Overall, weekly Carboplatin was better tolerated, inducing fewer severe toxicities than monthly Carboplatin.

### Carboplatin and paclitaxel efficacy

**Figure 2:**
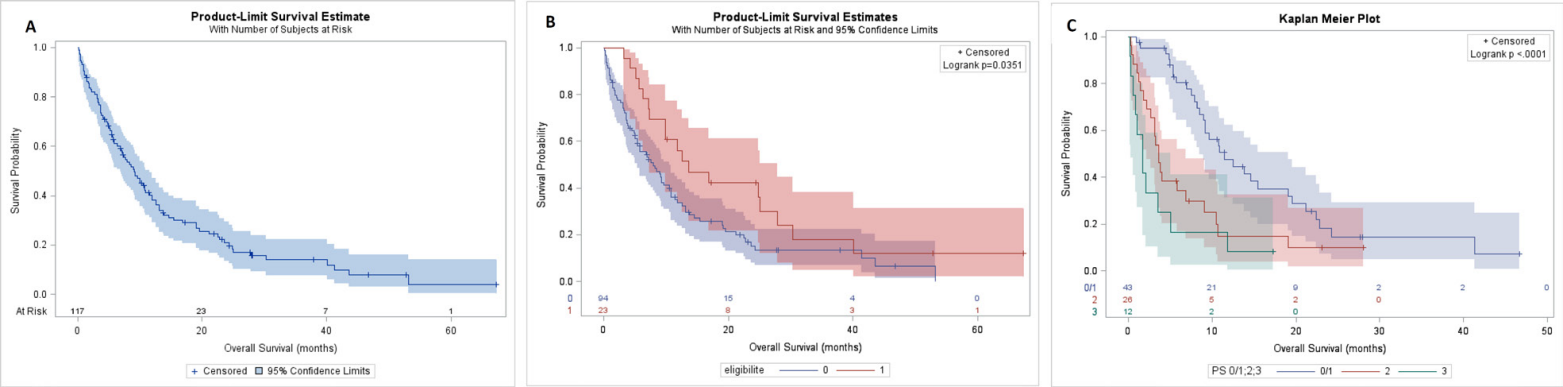
OS of population OS of all population (**A**), in eligible and no eligible group (**B**), in PS 0/1, 2 and 3 of no eligible group (**C**).

In intent to treat analysis the overall response rate was 40%, 5(4%) complete responses and 42 (36%) partial responses, whereas 33 (28%) patients were stabilized and 16 (14%) progressed. There were 21 (18%) unevaluable patients because they died before the first assessment. With a median of follow up of 28.1 months [95% CI; 24.3–52.8 months], the median OS for all population was 9.1months [95% CI; 6.9–11.5] (Figure 2A) and it was 13.7 months [95% CI; 7.3–27.9] and 8 months [95% CI; 5.4–10.7] for patients eligible to Cisplatin and not respectively (Figure 2B). Among patients ineligible for cisplatin, median OS was 11.5 months [95% CI; 8.5–19.1] if they were of PS0–1, but was 3.6 months [95% CI; 2.2–6.9] and 1.7 months [95% CI; 0.3–5.1] if they were of PS2 and 3 respectively (Figure 2C).

**Figure 3:**
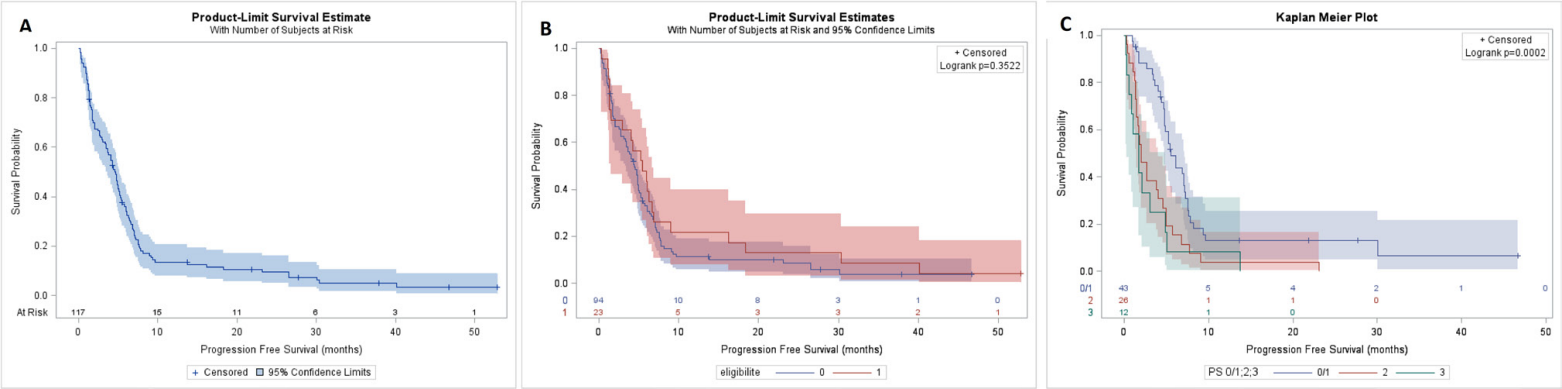
PFS of population PFS of all population (**A**), in eligible and no eligible group (**B**), in PS 0/1, 2 and 3 of no eligible group (**C**).

The median progression-free survival (PFS) for all population was 4.7months [95% CI; 3.7–5.2] (Figure 3A) and it was 5.5 months [CI 95%; 1.5–6.6] and 4.4 months [CI 95% ; 3.4–5.0] for patients eligible to Cisplatin and not respectively (Figure 3B). Among patients ineligible for Cisplatin, median PFS was 5.5 months [CI 95%; 4.8–7.1] if they were of PS0/1, but 2.0 months [CI 95%; 1.-4.0] and 1.7 months [CI 95%; 0.3–4.9] if they were of PS2 and 3 respectively (Figure 3C).

## DISCUSSION

The EXTREME (Platin-5FU-Cetuximab) regimen has become the standard of care in first-line therapy in patients with recurrent/metastatic head and neck squamous cell carcinoma with 36% responses and a median overall survival of 10.1 months [95% CI; 8.6–11.2] (versus 20% and 7.4 months [95% CI; 6,4–8,3] respectively for Platin-5FU) [10]. In subgroup analysis, OS was significantly better only with cisplatin at 10.6 months (vs 7.3 with chemotherapy alone) whereas there was not any significant difference with carboplatin (9.7 vs 8.3 months) suggesting that for patients ineligible to Cisplatin, the Carboplatin-EXTREME regimen is possibly not the best regimen.

Many patients aren’t eligible for Cisplatin and our study suggests Carboplatin with weekly Paclitaxel can be a good alternative for these unfit patients and even possibly for fit patients. Indeed the response rate of the whole population reaches 40% with a median OS of 13.7 months [95% CI; 7.3–27.9] and 8 months [95% CI; 5.4–10.7] for patients eligible and for patients ineligible to Cisplatin respectively. Evidently, the weak number of eligible patients (23) explains the large confidence interval forbidding definitive conclusion and does not authorize direct comparison with a prospective clinical trial. Further analysis are needed.

Again, even if a comparison isn’t possible between the results of a clinical trial and our retrospective study, Carboplatin and weekly Paclitaxel seems better for patients ineligible to Cisplatin with PS 0/1 compared to Carboplatin+5 Fluorouracil+Cetuximab in EXTREME study with a median OS of 11.5 months [95% CI; 8.5–19.1] vs 9.7 months and a median PFS of 5.5 months [CI 95%; 4.8; 7.1] vs 5.3 months. Survival was poor for patients with PS2 or 3 with OS of 3.6 and 1.7 months and PFS of 2.0 and 1.7 months respectively. Since this population is usually excluded from clinical trials we cannot draw definitive conclusion. Specific studies would be necessary to evaluate this population. Furthermore our definition of PFS was different than EXTEREME study, we considered the deaths only from cancer and not from any cause, it could be overestimated our results.

In our study, the tolerance of Carboplatin and weekly Paclitaxel appeared acceptable in this frail population with weekly Carboplatin regimen. In fact, the toxicities with monthly Carboplatin were more frequent and more severe. And a large part of the population of weekly Carboplatin group switched from monthly group after toxicities. Response rate of weekly Carboplatin group delivered from the beginning was slightly lower than for the overall population at 34% but we cannot conclude with only 29 patients. In further studies we propose to adopt a weekly regimen for both Paclitaxel and Carboplatin.

The main toxicities were hematologic with 26% and 3% anemia grade ≥3, 8% and 0% thrombopenia grade ≥3 and 40% and 28% neutropenia grade ≥3 in monthly and weekly Carboplatin group respectively.

Hepatotoxicity with Carboplatin and Paclitaxel is described in several studies [11, 14]. In our population, we didn’t observed any hepatic perturbation related to chemotherapy.

Toxicities of the Carboplatin group of the Extreme study are not available but in our practice it seems to be less toxic than Cisplatin, so we cannot formally compare our data.

Another study demonstrated safety and efficacy of weekly Carboplatin and Paclitaxel in 31 patients with locally advanced, distant metastases or recurrent head and neck squamous cell carcinoma. Median OS was 12.8 months [95% CI 8.6–15.5] and the major toxicity was hematologic too, with 22% neutropenia grade ≥3, 12% anemia grade ≥3 and 0% thrombopenia grade ≥3 [15].

With our results and since monotherapies with Paclitaxel, Cetuximab or Capecitabine are efficient after failure of platinum [16–18] there is sense to prefer bi-chemotherapy (by Carboplatin and Paclitaxel) to tri-chemotherapy (by Platin-5FU-Cetuximab) in order to allow sequential treatments that could increase survival [19]. Moreover, previous studies showed that Cetuximab could be combined with Paclitaxel with possible synergy and promising efficacy after failure of Platinum [6, 20] and thus could be better than EXTREME schedule for selected patients [21]. So, combination of the three agents Platin, Paclitaxel and Cetuximab could be of great interest. Indeed, a randomized phase II study in first-line reported 51.7% responses with Cisplatin-Paclitaxel-Cetuximab and an OS of 11 months [22]. Similarly, another phase II study combining Cisplatin Docetaxel and Cetuximab in first-line showed 44.4% responses and OS of 14 months [23].

After failure of Platinum, immunotherapy by checkpoint inhibitors has become the standard treatment. Indeed, the Checkmate 141 phase III randomized trial, showed that Nivolumab, a PD1 inhibitor, improved overall survival compared with single-agent therapy including Methotrexate, Docetaxel and Cetuximab [24] with a median overall survival of 7·5 months (95% CI, 5·5–9·1) and of 5·1 months (95% CI 4·0–6·0) (HR 0·70 [97·73% CI, 0·51–0·96]; *p* = 0·01) respectively. It is associated with fewer severe toxic effects and a better quality of life [25].

And currently, a phase III study compares Ipilimumab (CTLA 4 inhibitor) and Nivolumab with EXTREME schedule [26].

In conclusion, our study shows that weekly Carboplatin and Paclitaxel is a good option as first-line therapy for recurrent/metastatic head and neck squamous cell carcinoma, mostly for patients ineligible to Cisplatin. A future question would be the place of Cetuximab: in combination with Carboplatin and Paclitaxel or after failure of Nivolumab (or another anti-PD1 or PDL1). Similarly, combination of Carboplatin and Paclitaxel with immunotherapies could be relevant as in advanced non-small cell lung cancer [27, 28].

## MATERIALS AND METHODS

### Patient selection

We retrospectively reviewed the data in our institution between August 2009 and December 2016.

The inclusion criteria were as follows: (1) 18 or older patients with patients with histologically confirmed head and neck squamous cell carcinoma, (2) treated with Carboplatin and Paclitaxel in first-line for a recurrent or metastatic disease.

A first-line is defined by a first treatment in the recurrent/metastatic setting or by a switch from another treatment (due to toxicity of the first course) before any progression.

Prior treatment for a localized disease by surgery, radiotherapy or chemotherapy were permitted .

### Treatment

Different 28-days schedules were used. Paclitaxel was administered at 80 mg/m2 at day 1 (D1), day 8 (D8) and day 15 (D15). The standard schedule was Carboplatin AUC5 (area under the curve = 5) at D1 and in the alternative schedule Carboplatin was administered at AUC2 at D1, D8 and D15.

The doses could be reduced at the beginning or during treatment according to the patient’s frailty or to toxicities.

### Assessments

Response was evaluated every 6–8 weeks by repeated clinical and computed tomographic scan assessments on the basis of the extent of disease at presentation. Antitumor activity was evaluated according to the Response Evaluation Criteria In Solid Tumors criteria 1.1 [29].

### Statistical design

Overall survival (OS) was defined as the time from the date of first Carboplatin and Paclitaxel administration to the date of death.

Progression-free survival (PFS) was calculated from the date of first Carboplatin and Paclitaxel administration to the date of progression or death secondary to the cancer, whichever occurred first. If progression or death did not occur before the cut-off date, data were censored at the time of the last valid assessment.

Survival distributions were estimated by the Kaplan–Meier method.

## Abbreviations

RMHNSCC: recurrent/metastatic head and neck squamous cell carcinoma
D1: day 1
D8: day 8
D15: day 15
AUC5: area under the curve = 5
PS: performance status
PFS: progression free survival
OS: overall survival
